# Gel-Assisted Proteome Position Integral Shift Assay Returns Molecular Weight to Shotgun Proteomics and Identifies Caspase 3 Substrates

**DOI:** 10.1021/acs.analchem.4c02051

**Published:** 2024-08-07

**Authors:** Zhaowei Meng, Amir Ata Saei, Hassan Gharibi, Xuepei Zhang, Hezheng Lyu, Susanna L. Lundström, Ákos Végvári, Massimiliano Gaetani, Roman A. Zubarev

**Affiliations:** † Division of Chemistry I, Department of Medical Biochemistry and Biophysics, Karolinska Institutet, Stockholm 17177, Sweden; ‡ Chemical Proteomics Unit, Science for Life Laboratory (SciLifeLab), Stockholm 17165, Sweden; § Chemical Proteomics, Swedish National Infrastructure for Biological Mass Spectrometry (BioMS), Stockholm 17177, Sweden; ∥ Department of Microbiology, Tumor and Cell Biology, Karolinska Institutet, Stockholm 17177, Sweden; ⊥ HDXperts AB, Danderyd 18212, Sweden; # The National Medical Research Center for Endocrinology, Moscow 115478, Russia; ∇ Department of Pharmacological & Technological Chemistry, I.M. Sechenov First Moscow State Medical University, Moscow 119048, Russia; ○ Department of Pharmaceutical and Toxicological Chemistry, RUDN University, 6 Miklukho-Maklaya St., Moscow 117198, Russia

## Abstract

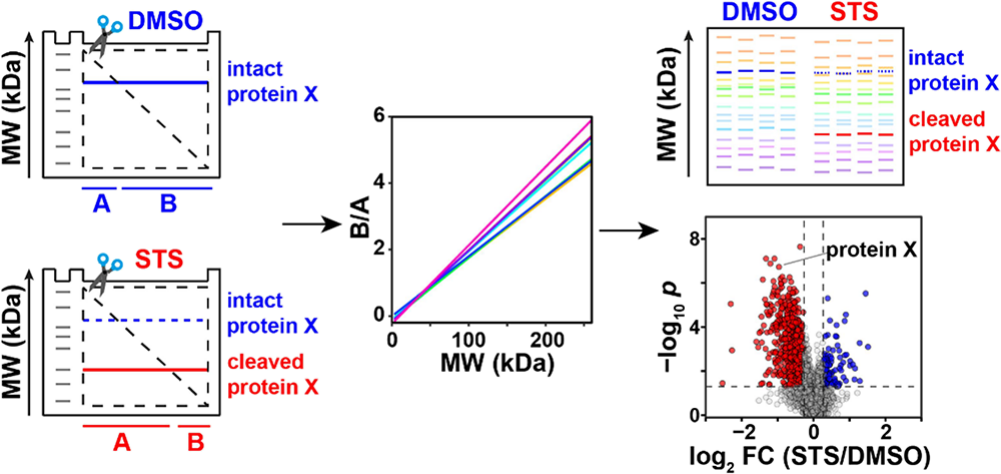

Here, we present a high-throughput virtual top-down proteomics approach that restores the molecular weight (MW) information in shotgun proteomics and demonstrates its utility in studying proteolytic events in programmed cell death. With gel-assisted proteome position integral shift (GAPPIS), we quantified over 7000 proteins in staurosporine-induced apoptotic HeLa cells and identified 84 proteins exhibiting in a statistically significant manner at least two of the following features: (i) a negative MW shift; (ii) an elevated ratio in a pair of a semitryptic and tryptic peptide, (iii) a negative shift in the standard deviation of MW estimated for different peptides, and (iv) a negative shift in skewness of the same data. Of these proteins, 58 molecules were previously unreported caspase 3 substrates. Further analysis identified the preferred cleavage sites consistent with the known caspase cleavages after the DXXD motif. As a powerful tool for high-throughput MW analysis simultaneously with the conventional expression analysis, the GAPPIS assay can prove useful in studying a broad range of biological processes involving proteolytic events.

## Introduction

Historically, proteomics was born as a gel-based analysis,^1−5^ relying primarily on protein separation by one-dimensional gel electrophoresis (1D-PAGE) and two-dimensional gel electrophoresis (2D-PAGE). 1D-PAGE, also known as sodium dodecyl sulfate-polyacrylamide gel electrophoresis (SDS-PAGE), involved the separation of proteins in a gel matrix based on their molecular weight (MW).^6,7^ Before being loaded onto a polyacrylamide gel, proteins are denatured by SDS and reduced by β-mercaptoethanol. When an electric current is applied, proteins migrate through the gel, with smaller proteins moving faster and farther, and larger ones traveling shorter distances on the gel. After electrophoresis, the gel is typically stained with Coomassie Blue or silver stain to visualize the separated protein bands. The MW scale is calibrated using a separate gel lane with a “ladder” of reference proteins with well-defined MW that exhibit narrow bands. When two conditions are compared, the bands of sample proteins, or rather proteoforms (the different protein variants of a given expressed gene product), that changed their position or density are excised, digested by trypsin and identified by mass spectrometry (MS). In contrast, in the GELFrEE strategy, the proteoforms are ultimately recovered at the end of separation in the solution phase,^8^ enabling simultaneous separation of proteome samples into 16 liquid fractions, covering the MW range of 10–150 kDa.^9^

2D-PAGE is a more advanced and powerful gel-based technique than 1D approaches as it offers better resolution of proteins and enables differentiation between their proteoforms. 2D-PAGE combines two orthogonal separation dimensions: isoelectric focusing (IEF) in the first dimension and SDS-PAGE in the second dimension.^10,11^ The resulting 2D gel image consists of a pattern of spots, each representing a different proteoform. In proteomics analysis, these spots are visualized, and the spots of interest (typically, the ones with altered position or density) are excised for identification by MS, while quantification is based on spot density.

Gel-based proteomics is time-consuming and labor-intensive and has several other limitations. One of the most serious drawbacks was the inability to identify more than a handful of shifted proteins. Density-based quantification is also a challenge. Despite these limitations, gel-based proteomics played a crucial role in the early days of proteomic research, being instrumental in cancer biomarker discovery,^12−14^ neurodegenerative disease research,^15−17^ drug development and pharmacology,^18,19^ and other important areas.

Subsequently, gel-based methods were replaced by “shotgun” or “bottom-up” proteomics,^20,21^ in which the S–S bonds in proteins are first reduced and alkylated, the proteome is digested with trypsin, and the peptide mixture undergoes liquid chromatography tandem mass spectrometry (LC-MS/MS) analysis. Compared to gel-based proteomics, the shotgun approach has several clear advantages, such as the depth and breadth of analysis as well as the use of several independently obtained peptide abundances for protein quantification. However, the loss of MW information is an indisputable drawback in the bottom-up approach. This information is particularly important in studying the appearance of abnormal protein fragments, including truncated or cleaved proteins, especially in the context of cell death,^22,23^ cancer,^24,25^ and other diseases.^26,27^

In the shotgun approach, detection of protein cleavage is not straightforward and it is not part of the normal analysis workflow. One indication of such a cleavage is the missing tryptic peptide encompassing the cleavage site. However, the sequence coverage of most proteins in a typical shotgun proteomic analysis is less than 50%,^28^ and missing peptides are a common occurrence. Another possible indication is the presence of semitryptic peptides. However, semitryptic peptides are usually ignored in shotgun proteomics as their true positive identification involves a significantly higher burden of proof than that of fully tryptic peptides.^29^

As an SDS-PAGE gel would reveal the MW shift in the case of a protein cleavage, a method emerged termed the protein topography and migration analysis platform (PROTOMAP)^30^ in which the 1D-PAGE gel with the separated proteome is cut into a large number *N* (20 ≤ *N* ≤ 100) of narrow bands, with the proteins in each band digested and analyzed separately by LC-MS/MS. The PROTOMAP approach demonstrated its analytical utility by detecting MW shifts in proteins undergoing proteolytic cleavage by caspases and identifying caspase substrates by such shifts.^30^ PROTOMAP has also revealed numerous proteolytic events in blood plasma, providing significant coverage of the coagulation degradome.^31^ However, to achieve sufficient statistical power, the method required the already large number of gel bands to be analyzed in numerous replicates, resulting in time-consuming sample preparation and a vast number of LC-MS/MS analyses. This ultimately prevented PROTOMAP from becoming a standard method.

**Figure 1:**
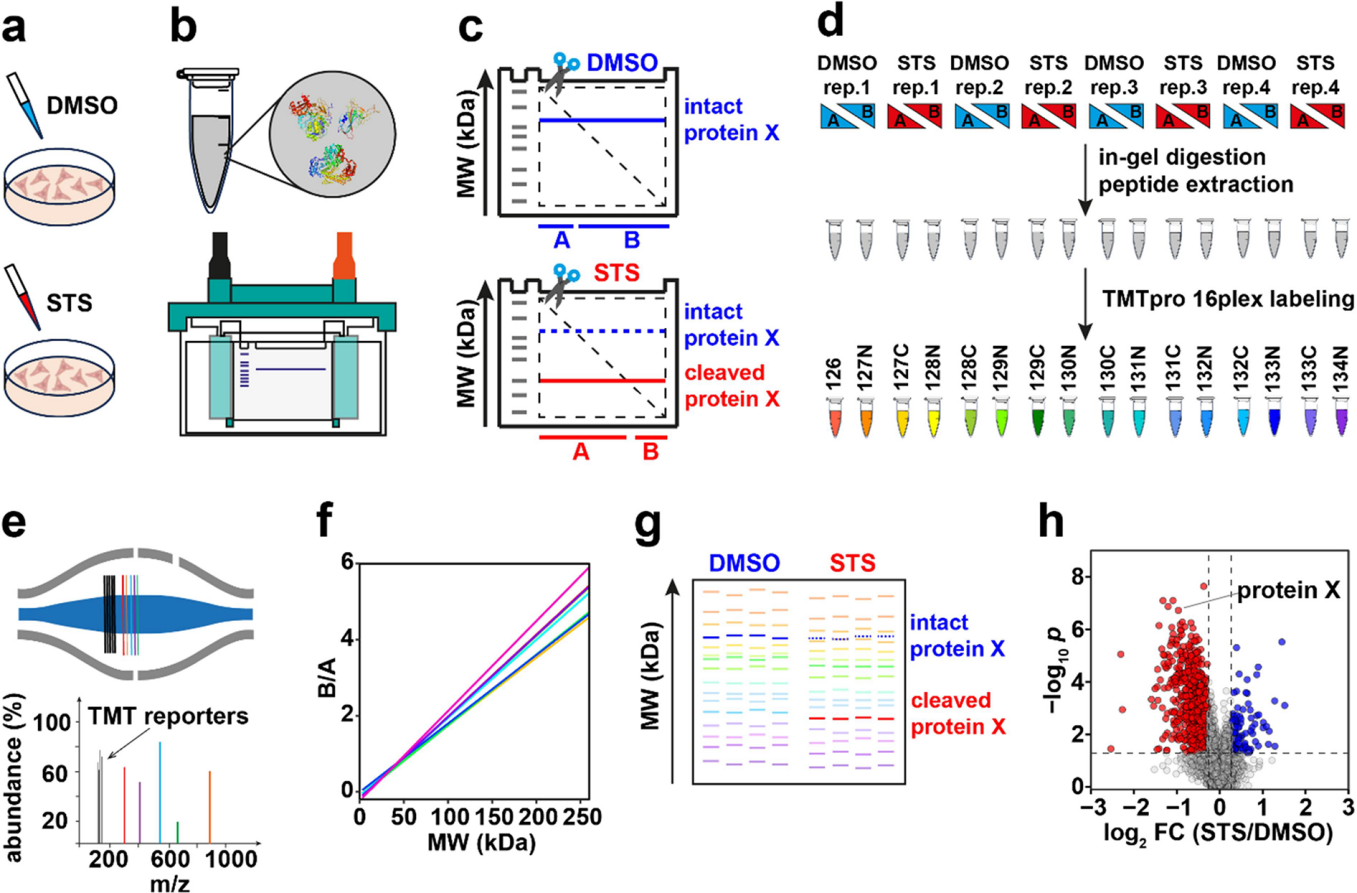
GAPPIS workflow. (a) HeLa cells are treated with staurosporine (STS) or DMSO (control); (b) cell lysis and SDS-PAGE. (c) Each gel is cut diagonally; the abundance ratio B/A provides the position (MW) of the proteins. A cleavage in protein X results in a decrease in its MW and B/A ratio. (d) Gel pieces are digested in-gel, and then peptides are extracted and TMT-labeled followed by fractionation. (e) LC-MS/MS analysis. (f) B/A ratio is converted to MW, providing MW scale calibration using selected proteins and MW estimation for all other proteins. (g) A pseudo gel shows proteins’ MW estimations and their changes between DMSO- and STS-treated HeLa cells. (h) A volcano plot identifies proteins with significant MW shifts.

Here, we present the gel-assisted proteome position integral shift (GAPPIS) assay as a high-throughput alternative to PROTOMAP. GAPPIS derives the MW information on thousands of proteins by analyzing only two gel pieces. To achieve this, each gel lane is cut diagonally along the whole length, providing pieces A and B (Figure 1a–c). Each protein band therefore becomes split into two parts. The proteins are then extracted from both gel pieces, reduced, alkylated, and then digested. The digests of the extracted proteomes are thereafter labeled by tandem mass tag (TMT), and the TMT-multiplexed samples are analyzed by LC-MS/MS (Figure 1d–e). It is clear from Figure 1c that a protein with a higher MW exhibits a higher ratio between the protein abundances in pieces B and A and vice versa. Thus, the GAPPIS ratio (B/A) will provide an estimate of the protein MW, while the sum (A + B) will reflect the overall protein abundance of each specific protein in the proteome. For more precise MW scale calibration, well-known proteins with minimum post-translational modifications (PTMs) should be used (Figure 1f).

To test the GAPPIS assay performance, we employed a biological system similar to that in the first PROTOMAP study.^30^ Namely, the pan-kinase inhibitor staurosporine (STS) was used to treat HeLa cells, which induced apoptosis and activated caspase 3 proteolytic cleavages. Our goal was to identify the substrates of caspase 3 and compare the GAPPIS results with the three previous works on the subject.^30,32,33^ In one study,^30^ 261 caspase 3 substrates were identified by PROTOMAP. Prior to that, Lüthi and Martin have compiled the CASBAH database containing all known by then caspase substrates (313 human proteins in total).^32^ Subsequently, Mahrus et al. selectively biotinylated free protein N-termini, performed enrichment of the corresponding N-terminal peptides, and identified 282 caspase 3 substrates.^33^ The overlap between the caspase substrate lists in these studies ranges between 29 and 42%, which testifies to the need for additional research on the subject. With GAPPIS, we confirmed previously found caspase substrate candidates and validated new caspase 3 substrates.

## Experimental Section

Detailed experimental information including the cell culture, cell lysis, SDS-PAGE, gel excision, proteomic sample preparation, high pH fractionation, LC-MS/MS analysis, and data analysis is provided in the Supporting Information.

### Cell Work and GAPPIS Sample Preparation

HeLa cells were treated in four biological replicates with 300 nM staurosporine (STS) or vehicle (DMSO) for 4 h. Cell lysate was loaded onto NuPAGE 4–12% Bis-Tris Mini Protein Gel with two wells for electrophoresis. One STS-treated sample and a DMSO-treated sample were processed in the same gel tank. After electrophoresis, the gels were washed and excised diagonally into two parts (A and B), after which each part of the gel was cut into 1 × 1 mm cubes and transferred into 5 mL LoBind tubes. The samples were reduced, alkylated, and in-gel digested. The digestion solution with extracted peptides was cleaned up for subsequent TMT labeling. After cutting each gel lane into two pieces A and B, four replicates of DMSO- and STS-treated HeLa cells (eight samples) produced 16 subsamples, and one set of TMTpro 16plex was used for labeling these 16 subsamples, as shown in Figure 1g. All 16 TMT-labeled samples were combined, desalted, and then underwent high-pH fractionation.

### LC-MS/MS Analysis

The sample fractions were analyzed on an Orbitrap Fusion Lumos mass spectrometer equipped with an EASY Spray Source and connected to an Ultimate 3000 RSLC nano UPLC system (all, Thermo). Injected samples were loaded on a 2 cm long C18 nano trap column and separated on an EASY Spray C18 nano LC column (Acclaim PepMap RSLC; 50 cm × 75 μm). To identify and quantify TMT-labeled peptides, we utilized both the MS2 and SPS MS3 methods.

### Data Processing

The raw LC-MS/MS data were analyzed by MaxQuant (version 2.2.0.0) using the Andromeda search engine against the UniProt Human proteome database (20,607 human sequences). Enzyme specificity was trypsin, with a maximum of two missed cleavages permitted. When needed, a semitryptic option was specified for the identification of semitryptic peptides in the MS2 data set. Cysteine carbamidomethylation was set as a fixed modification, while methionine oxidation, N-terminal acetylation, and asparagine or glutamine deamidation were used as a variable modification. A false discovery rate (1%) was used as a filter at both protein and peptide levels. Default settings were employed for all of the other parameters. Peptide quantification was executed by using a TMTpro 16plex. The detailed post-MaxQuant data analysis is described in the Supporting Information.

## Results

### Establishing MW Scale

**Figure 2:**
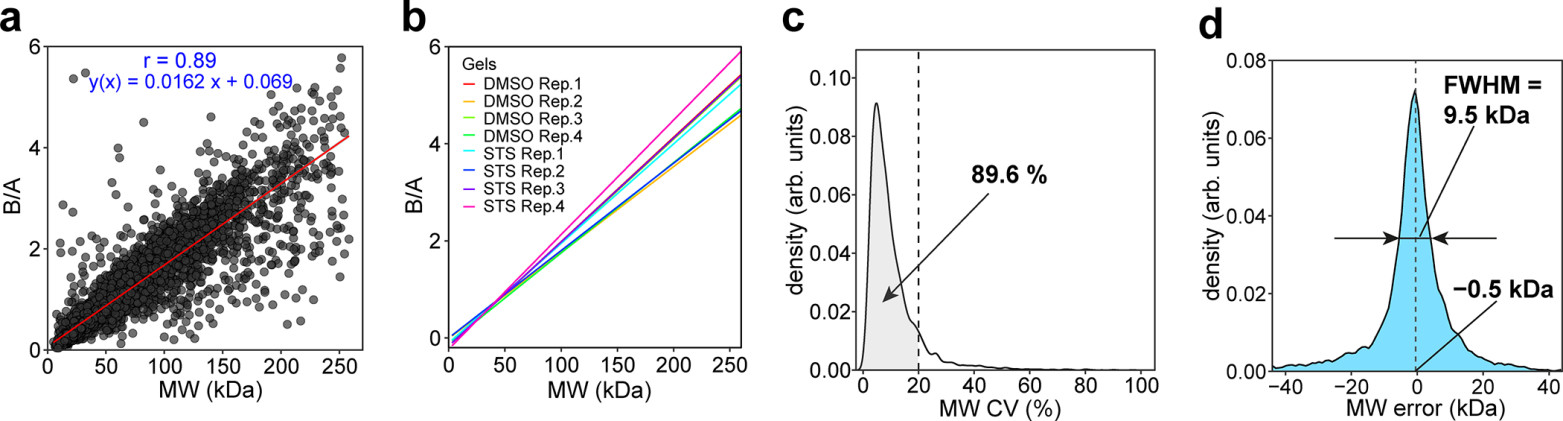
Correlation of the protein GAPPIS ratio (B/A) with MW for the MS3 data set. (a) Correlation of B/A ratios with MW for all 6640 identified proteins. (b) Calibration curves for all eight gels. (c) CV distribution of the protein B/A-estimated MWs. (d) Error distribution of the B/A-estimated MWs.

In the MS2-based analysis, we identified and quantified 7433 proteins based on 103,927 peptides; the corresponding values for the MS3 data set were 6640 proteins and 69,843 peptides. Figure 2a demonstrates a strong linear correlation (*R* = 0.89) observed in the MS3 data set between the protein B/A value and theoretical MW in the range between 3 and 260 kDa. In the MS2 data set, the correlation was also significant (Figure S3) but weaker than in MS3. As the precision of MW determination is defined by the precision of abundance measurements in pieces A and B, it was not surprising that the MS3 approach provided better results than MS2 due to the reduction of the peptide cofragmentation.^34^ As MS2 data were still usable, we also employed them for further analysis.

To establish the robust MW calibration lines, we selected in the MS3 data set 641 reference proteins according to the following criteria: (a) not to be listed as caspase substrates in either of the three previous studies,^30,32,33^ (b) have a sequence coverage of ≥50%, (c) be devoid of UniProt-reported PTMs, not to be part of mitochondrion, contain a transit peptide, or being a repressor,^35^ and (d) the B/A values of all peptides should have CV <30% across the four replicates. The resultant lines are shown in Figure 2b. All plots demonstrated strong correlations (*r* > 0.95) between B/A and MW (Figure S1).

Indeed, if the protein band position Y on a gel is a linear function of MW, then Y = a*MW, where *a* is a proportionality factor. The band is split into two parts, A and B, such as A + B = 1. As the cut is diagonal, B is proportional to Y, and B = b*Y, where *b* is another proportionality factor. Then, B = b*a*MW, and A = 1 – b*Y = 1 – b*a*MW. Therefore, B/A = (b*a*MW)/(1 – b*a*MW) = (1 – 1 + b*a*MW)/(1 – b*a*MW) = (1 – [1 – b*a*MW])/(1 – b*a*MW) = 1/(1 – b*a*MW) – 1. As 0 < B = b*a*MW < 1, one can employ for approximation Taylor’s expansion, 1/(1 – *x*) = 1 + *x* + *x*^2^ + *x*^3^..., and ignore higher order terms starting from quadratic. Thus, B/A ≈ 1 + b*a*MW – 1 = b*a*MW. Therefore, in the first approximation, B/A is proportional to MW. One should however keep in mind that this back-on-the-envelope calculation is only valid if the protein band position Y on a gel is a linear function of MW, while in reality, it may be closer to a logarithmic function.^36^ Yet, our numerical analysis (data not shown) demonstrated that even in this case, the approximation B/A ≈ k*MW is satisfactory.

The linear regression between the GAPPIS ratio (B/A) and MW of the reference proteins was used to estimate MW for all other detected proteins. Overall, 89.6% of all proteins exhibited CV between replicate MW estimates of less than 20%, with a peaked frequency of CV at only 4.9% (Figure 2c). The deviations of the estimated MW from the theoretical value (without any PTMs) form a sharp bell-like distribution (Figure 2d) centered at −0.5 kDa and with a full width at half-maximum (fwhm) of only 9.5 kDa. A similar analysis was conducted on the MS2 data set, yielding somewhat less accurate results (Figures S2 and S3). Note that in the original PROTOMAP study, the gel was cut into 22 pieces, which for the range of 0–250 kDa corresponds to a MW resolution of ≈11 kDa.^30^ Therefore, the GAPPIS approach is at least as precise as PROTOMAP in MW estimation, despite being based on the analysis of just two samples instead of 22.

**Figure 3:**
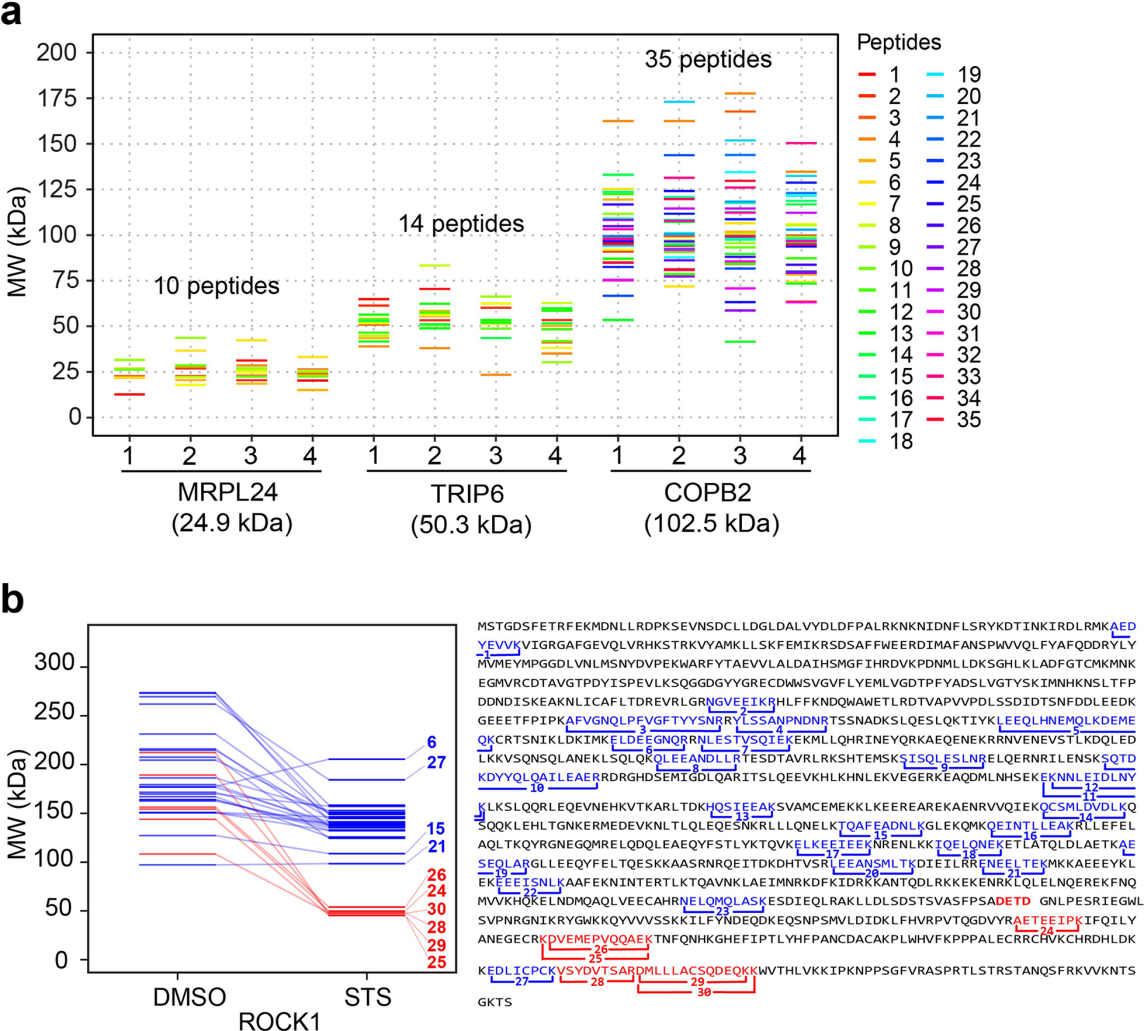
Protein MW estimation from peptide B/A values. (a) Pseudogel with three proteins’ MW distributions across four replicates in DMSO-treated HeLa cells. The color coding of the peptides is shown on the right of the plot. (b) Left: A pseudogel with peptide-derived MW estimations of the caspase 3 substrate ROCK1 in DMSO- and STS-treated HeLa cells. Right: mapping of the identified peptides (numerated 1–30) on the ROCK1 sequence.

Since protein MW information is derived in GAPPIS from each peptide independently, it is worth carefully investigating the distribution of the peptide-level data. For that, we selected three proteins with theoretical MW of 24.9, 50.3, and 102.5 kDa, which were not included in the calibration set. For a low-mass protein, the peptide MW data are not surprisingly localized tightly around the protein’s theoretical MW, while the spread between the peptides increases with MW (Figure 3a). Besides a longer traveling path on a gel, high MW proteins are more likely to have multiple proteoforms, which explains this result. However, higher mass proteins also produce a larger number of peptides, and therefore despite the data spread for individual peptides, the center of gravity of the peptides’ positions is rather stable with respect to MW. Analysis showed that the median of the peptide’s MW data is more robust than the average value, likely because medians tend to ignore statistical outliers that more often are false positives. For all three chosen proteins, the deviation of the median-derived MWs from the theoretical MW values did not exceed 5 kDa, underscoring the precision of the GAPPIS approach.

It should be noted that the gels are not perfect MW analyzers as the protein position on the gel is determined by protein mobility that in turn is defined by the protein net charge and its molecular radius as well as amino acid composition.^37^ The net charge is determined by the sum of the basic and acidic amino acids in the protein, and the molecular radius is determined by the protein’s tertiary structure. Therefore, systematic deviations of some proteins from the calibration curve should be expected. This deviation is not specific to GAPPIS and affects all gel-based approaches. Yet, for many practical purposes, the MW estimate from gels is quite sufficient.

Upon establishing the MW scale, we applied GAPPIS analysis to the identification of caspase 3 substrates. To that end, we applied four complementary techniques: MW shift, semitryptic peptide analysis, novel standard deviation analysis, and skewness shift analysis. As the results from these techniques were statistically independent (central moments of distribution are mathematically orthogonal), an intersection of the proteins supported by at least two techniques was considered to be their validation. We also compared the identified caspase 3 substrate candidates with those reported earlier in literature and analyzed the preferred motifs of the cleavage sites to verify their origin from caspase activity.

### MW Shift Analysis

As an example, for the protein ROCK1 (theoretical MW 158.2 kDa), a well-established substrate for caspase 3 in STS-treated HeLa cells,^38,39^ 30 peptides were identified in the GAPPIS analysis (sequence coverage 32.9%). The median MW of all these peptides shifted from 173.9 ± 17.9 kDa for the untreated sample to 135.2 ± 7.4 kDa for the STS-treated one (*p* < 0.05), immediately identifying this protein as a protease substrate. The known caspase 3 cleavage in ROCK1 produces an N-terminal fragment of 130 kDa and a C-terminal fragment of 28 kDa in size, with the cleavage site located after the sequence DETD_1113_.^40^ In GAPPIS analysis of STS-treated HeLa cells, most of the 24 peptides mapping to the N-terminal cleavage fragment (highlighted in blue in Figure 3b) are tightly clustering around MW 141 ± 20 kDa, with all six C-terminal peptides (highlighted in red) clustering around 48 ± 3 kDa. From those data, the cleavage position could be located between residues K_1083_ (at the C-terminus of the detected N-terminal peptide NELQMQLASK) and A_1187_ (at the N-terminus of the C-terminal peptide AETEEIPK). We could establish the precise location of the cleavage site by semitryptic peptide analysis (see below).

**Figure 4:**
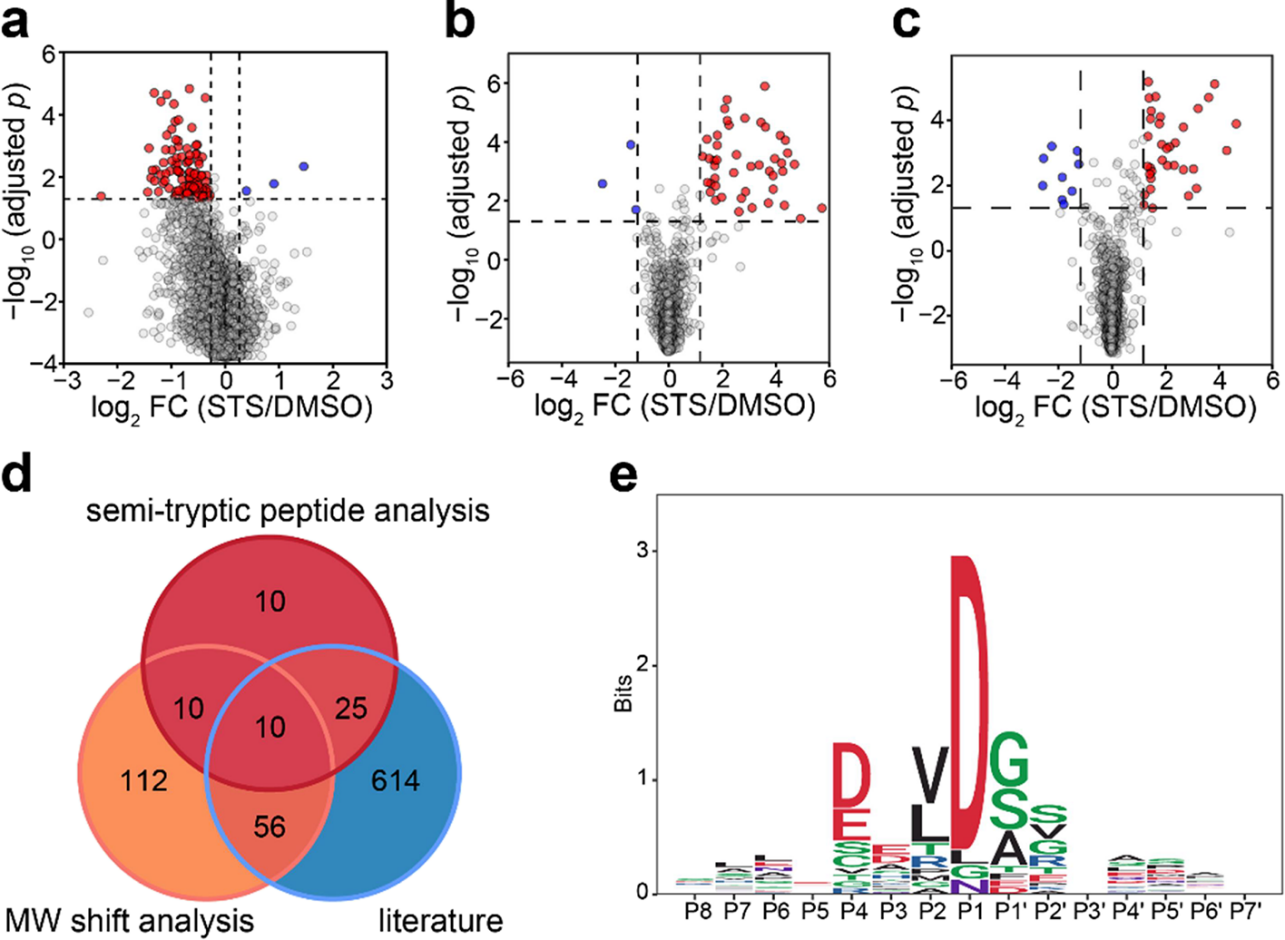
GAPPIS MS3 data identified proteins with a significant MW shift. (a) Volcano plot with 102 proteins significantly shifted to lower MW (red), and 3 proteins significantly shifted to higher MW (blue). (b) Volcano plot of semitryptic peptides with K/R as the last amino acid residue cells with a significant increase (red) and decrease (blue) in the abundance ratio to fully tryptic peptide STS-treated HeLa cells. (c) Same for semitryptic peptides with K/R before the first amino acid residue. (d) Overlap of the GAPPIS-identified caspase 3 substrates with the three previous studies. (e) Preferred sequence motif for the cleavage sites in 24 semitryptic peptides.

On a volcano plot, there are many more proteins with a significant decrease in MW upon STS treatment compared to increased MW (102 vs 3; Figure 4a). This was expected as the proteolytic activity is the major process in apoptosis. If all the proteins showing increased MW were false positives (while in reality, some positive shifts could be due to PTMs), then the false discovery rate (FDR) of GAPPIS analysis can be roughly estimated as ≤3%, which is remarkably low compared to the alternative approaches. A similar analysis of the MS2 data set identified 155 proteins with a significant decrease in MW (Figure S4), of which 69 (68%) were the same as with the MS3 approach. There were 25 proteins with increased MW, which estimated the FDR in the MS2 data set to be 13.8%. Merging these two data sets led to the identification of 188 potential caspase 3 substrates by a negative MW shift. Of these, 66 proteins (35%) overlapped with at least one of the three previous studies, while the expected random overlap is 16 proteins. At the same time, the MW of 26 proteins shifted positively. If all these were false positives, then the FDR in the merged data set would be estimated at around 12%. If, however, there is a strong reason to suspect a PTM presence (e.g., in the case of the use of glycosylating enzymes^41^), then a broader database search including probable PTMs as variable modifications could verify the hypothesis that the MW shift is due to PTMs.

### Semitryptic Peptide Analysis

Reliable identification of the cleavage site by semitryptic peptides is not an easy task as the space of possible sequences increases by an order of magnitude compared to fully tryptic peptides, with the FDR increasing proportionally. To enhance confidence in the identified semitryptic peptides, we introduced additional requirements as a filter for false discoveries. One such requirement was that the semitryptic peptide abundance should increase significantly after STS treatment. Another requirement was that the fully tryptic peptide partially overlapping with a given semitryptic peptide should also be present in the data set. We identified 44 semitryptic peptides with a classical tryptic C-terminus that were both paired with their corresponding fully tryptic partner as well as showed a significant increase in their abundance ratio to the tryptic counterpart in the STS-treated HeLa cells compared to DMSO-treated cells (Figure 4b). A similar analysis was conducted for semitryptic peptides with a classical tryptic N-terminus, resulting in the identification of 34 such molecules (Figure 4c). After merging these results, we identified as caspase 3 substrate candidates a total of 55 proteins in which semitryptic peptides increased their abundance after treatment compared to their fully tryptic counterparts and 9 proteins with decreased semitryptic peptide abundance (if all are false positives, then FDR ≈ 14%). Among our caspase 3 substrate candidates, 35 proteins were found in the literature, giving a record 64% overlap with previous research (Figure 4d), while a random match would produce only five overlapping proteins on average.

Analysis of the preferred cleavage motif was performed using 23 semitryptic peptides satisfying the following conditions: (a) paired with their fully tryptic counterparts; (b) showing a significant increase in the abundance ratio of a semitryptic to a fully tryptic peptide in STS-treated HeLa cells; (c) stemming from the GAPPIS-identified proteins exhibiting a significant negative MW shift in Figure 4a and Figure S4. The resultant pattern shown in Figure 4e revealed that cleavages consistently occurred after the DXXD motif, in line with the known caspase cleavage preference.^33,42,43^ This finding further validated the selected proteins as caspase substrates.

### Standard Deviation (SD) Analysis

**Figure 5:**
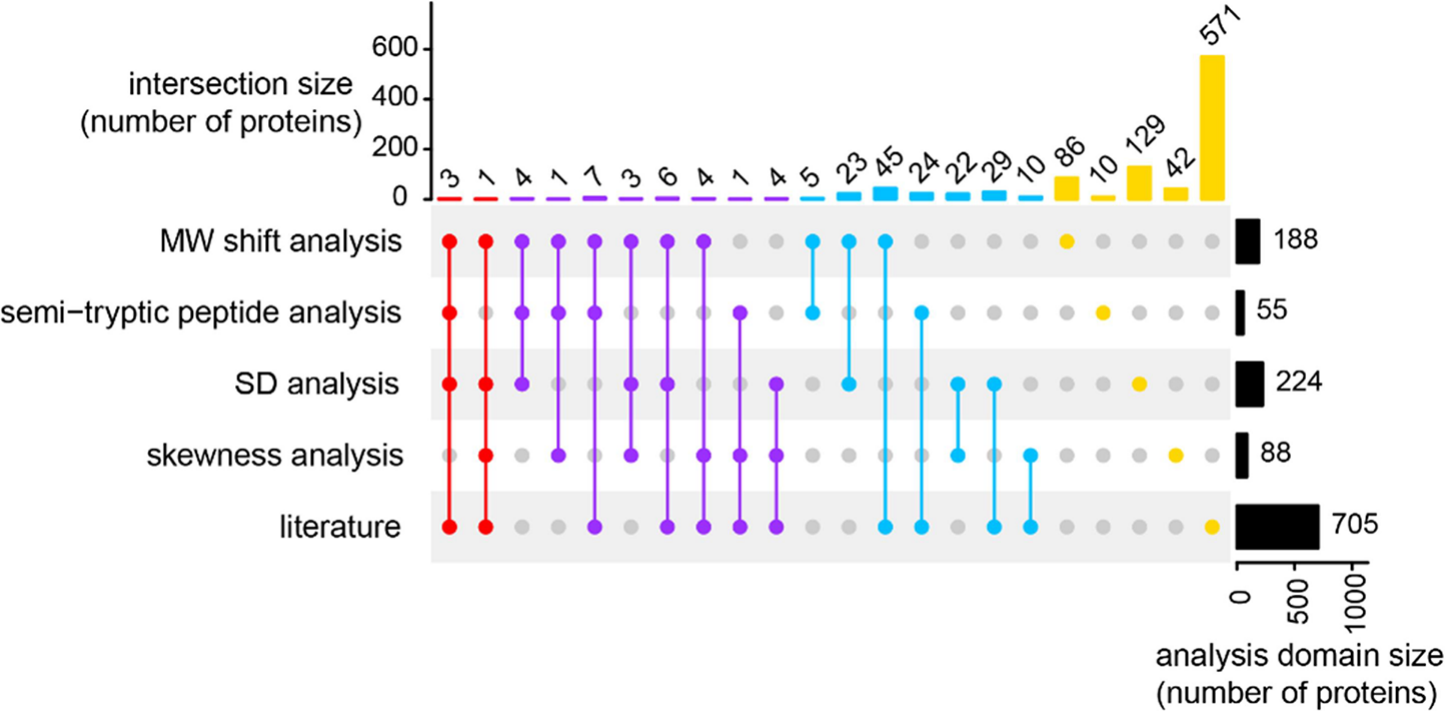
Validation analysis. Overlap between the four independent analysis domains as well as published literature for the GAPPIS-revealed caspase 3 candidate substrates.

As each peptide carries in GAPPIS information on the MW of the protein it belongs to, we used standard deviation (SD), the second central moment of statistical distribution, as a metric for assessing the dispersion of each protein’s peptide-to-peptide MW estimate. The SD of protein MW exhibited a nonlinear upward trend with MW increasing, following the empirical formula SD = 0.086MW^1.29^ (residual standard error 13 kDa) for DMSO treatment and SD = 0.086MW^1.23^ (residual standard error 10 kDa) for STS treatment (Figure S5a,b). As the protein MW decreases after cleavage, we expect a reduction in SD for MW of the substrate proteins. In agreement with this expectation, among the 3384 proteins identified with ≥7 peptides (such a peptide number threshold was required for reliable SD estimation), we observed 224 proteins exhibiting a significantly decreased SD after STS treatment compared to DMSO-treated controls (Figure S5c). At the same time, only 26 proteins displayed a significant increase in SD, which corresponds to an ≈10% FDR if all of the latter proteins were false positives. Out of these 224 proteins, 40 molecules (18%) overlapped with at least one of the three previous studies (Figure 5), while a random match would give 19 proteins on average. Additionally, the pseudo gel for protein Q6WCQ1 with altering SD (Figure S6a) revealed that its MW significantly decreased from 126.8 ± 71.4 to 49.7 ± 13.3 kDa after STS treatment.

### Skewness Analysis

Skewness is the third central moment of a statistical distribution and is responsible for its asymmetry. Seeking to fully utilize the wealth of information obtained in the GAPPIS approach, we tested whether skewness analysis could be a complementary method of protease substrate identification. The basic assumption was that the peptide MW data distribution of an intact protein should be almost symmetric on the MW scale, being centered around the protein MW, while the presence of two unequal fragments after cleavage would result in an asymmetric distribution skewed on the lower-mass side (negative skewness shift). As an example, Figure S6b shows a pseudogel for three proteins with positive, negative, and nearly zero skewness shifts. Consistent with the above assumption, among the 1569 proteins identified with ≥13 peptides (high threshold needed to obtain precise skewness estimate), we found 88 proteins with significantly decreased skewness and 25 proteins (likely false positives, FDR ≈ 22%) with significantly increased skewness after STS treatment compared with DMSO-treated controls (Figure S7).

### Validation Analysis

As indicated earlier, we considered an overlap between any two of the above complementary analysis domains to be a validation for the caspase 3 candidate substrates. In total, 84 proteins were validated, while the expected random overlap would give less than eight proteins on average. Of these 84 proteins, 12 proteins were validated by three or more domains, and 26 molecules (31%) were found in the literature (Figure 5 and Table S1). Therefore, GAPPIS provided 58 new caspase 3 substrates.

## Conclusions

Here, we introduced GAPPIS, a novel shotgun proteomics approach that, being a virtual top-down analysis, brings back the MW information to proteomics. This information, which is orthogonal to both protein abundance and solubility, is obtained from significantly fewer LC-MS/MS analyses than are required by the original gel-based PROTOMAP approach, being at least as precise in terms of MW estimation. To be fair, GAPPIS gel pieces contain significantly more complex samples, requiring more fractions to be analyzed to reach the same proteome depth. Also, should the same protein appear in several bands with distinctly different MWs (e.g., due to multiple proteoforms), the PROTOMAP approach would have had a better chance than GAPPIS of differentiating this from a single MW situation. However, by applying standard deviation analysis, GAPPIS could still potentially detect an unexpectedly broad distribution of MW data from the peptides belonging to this protein. Another limitation of the method is that it detects only complete or near complete proteolytic truncations, while low-occupancy cleavages may remain unnoticed.

The “killer application” of GAPPIS seems to be the same as PROTOMAP, i.e., the identification of protease substrates in living cells. In this application, GAPPIS can be a competitor to existing techniques, such as, e.g., N-terminomics.^33,44^ There are hundreds of proteases in mammalian cells, implicated in all kinds of biological processes, and the knowledge of their substrates and specificity is important, not least because they represent potential drug targets.^45,46^ With GAPPIS, this information may become much more easily available. More importantly, the MW information is encoded in every peptide, both tryptic and semitryptic. The wealth of this information is hard to fully appreciate from the standpoint of conventional shotgun proteomics, and in this work, we are just scratching the surface of potential new applications. It is however evident that besides the first central moment (centroid) of the peptide MW distribution, useful information can also be found in the second (standard deviation) and, possibly, in the third (skewness) moment.

As a final comment, the MW information comes in GAPPIS from the precisely measured peptide abundances in gel pieces A and B. The more precise the abundance measurements, the better the MW estimation. We found that the MS3-based TMT quantification provides superior performance compared to the easier, more sensitive, and much more widely used MS2-based quantification. This finding should encourage further progress in MS instrumentation.

## Supporting Information Available

Supplementary figures and table: detailed description of experimental procedures and methods including cell culture, GAPPIS sample preparation, proteomic sample preparation, parameters for LC-MS/MS, and data analysis (PDF)
