# Supplementary material for: Gel-Assisted Proteome Position Integral Shift Assay Returns Molecular Weight to Shotgun Proteomics and Identifies Caspase 3 Substrates
Source: Anal Chem. 2024 Aug 7;96(33):13533–41. doi: 10.1021/acs.analchem.4c02051 (PMC11339726; doi:10.1021/acs.analchem.4c02051)
Supplement: Supplementary file 1 — ac4c02051_si_001.pdf [file ac4c02051_si_001.pdf]

Gel-Assisted Proteome Position Integral Shift (GAPPIS) Assay Returns Molecular Weight to Shotgun Proteomics and Identifies Caspase 3 Substrates

Supporting Information

Zhaowei Meng[1,2,3], Amir Ata Saei[4], Hassan Gharibi[1,2,3], Xuepei Zhang[1,2,3], Hezheng Lyu[1,5], Susanna L. Lundström[1,2,3], Ákos Végvári[1], Massimiliano Gaetani[1,2,3], Roman A. Zubarev[1,3,6-8*]

[1] Division of Chemistry I, Department of Medical Biochemistry and Biophysics, Karolinska Institutet, 17177, Stockholm, Sweden

[2] Chemical Proteomics Unit, Science for Life Laboratory (SciLifeLab), 17165, Stockholm, Sweden

[3] Chemical Proteomics, Swedish National Infrastructure for Biological Mass Spectrometry (BioMS), 17177, Stockholm, Sweden

[4] Department of Microbiology, Tumor and Cell Biology, Karolinska Institutet, 17177, Stockholm, Sweden

[5] HDXperts AB, 18212, Danderyd, Sweden

[6] The National Medical Research Center for Endocrinology, 115478 Moscow, Russia

[7] Department of Pharmacological & Technological Chemistry, I.M. Sechenov First Moscow State Medical University, 119048, Moscow, Russia

[8] Department of Pharmaceutical and Toxicological Chemistry, RUDN University, 6 Miklukho-Maklaya St, Moscow, 117198, Russia

Table of Contents

Supplementary Figures S1-S7 (page S2-S8)

Supplementary Table S1 (page S9-S11)

Methods (page S12-S16)

Cell Work and GAPPIS Sample Preparation (page S12-S13)

LC-MS/MS (page S14-S15)

Data Processing (page S15-S16)

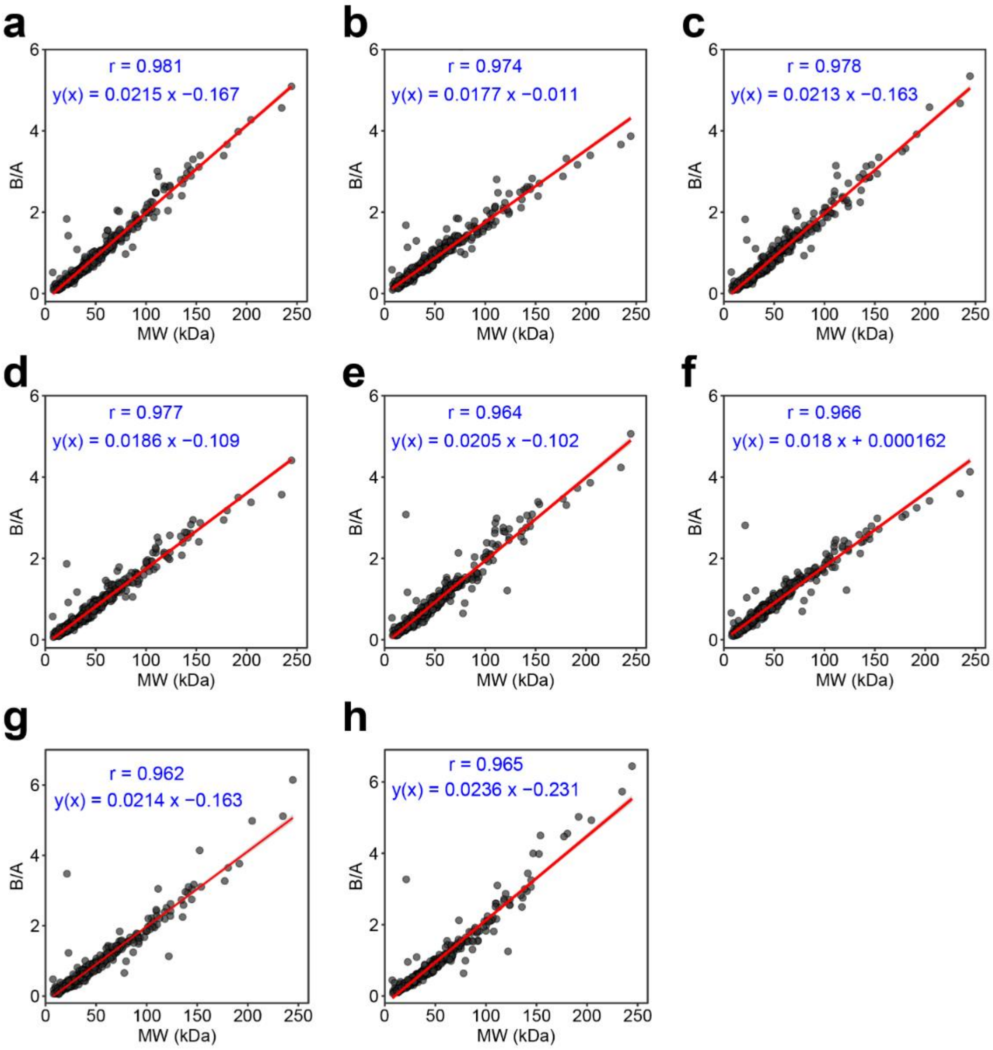

Figure S1. Calibration curves converting B/A values to MW for the MS3 data set. (a) to (d) Data calibration curves for the four replicates of DMSO-treated HeLa cells, respectively. (e) to (h) Data calibration curves for the four replicates of STS-treated HeLa cells, respectively.

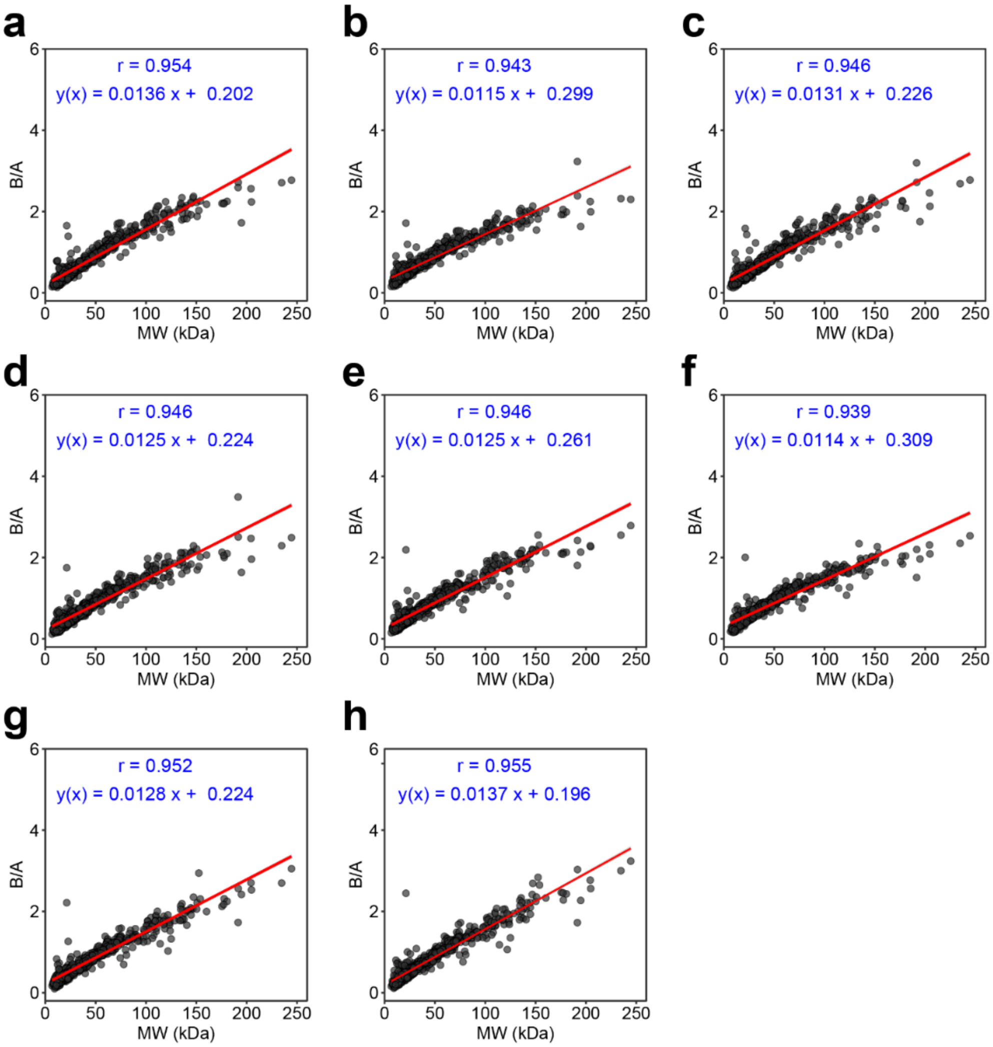

Figure S2. Calibration curves converting B/A values to MW for the MS2 data set. (a) to (d) Data calibration curves for the four replicates of DMSO-treated HeLa cells, respectively. (e) to (h) Data calibration curves for the four replicates of STS-treated HeLa cells, respectively.

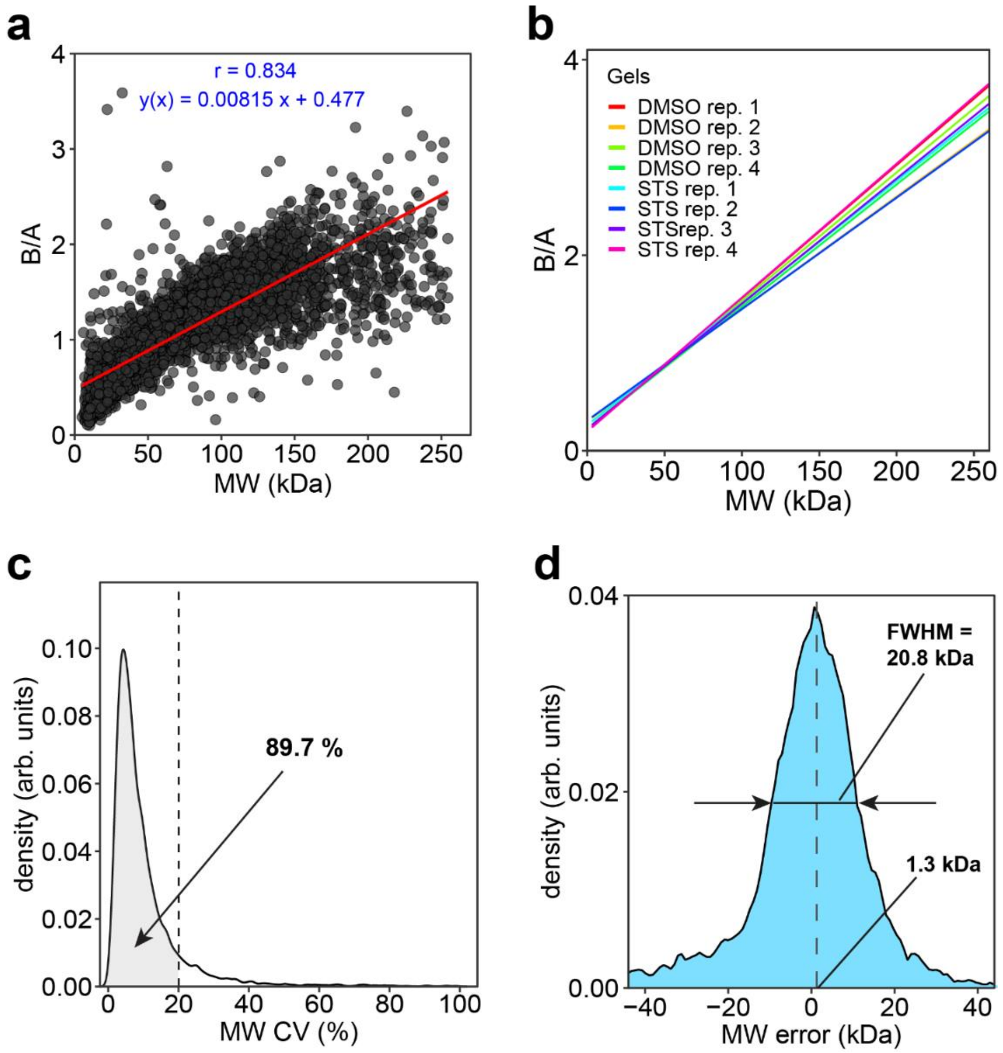

Figure S3. Protein MW estimations from peptide B/A ratios for the MS2 data set. (a) Correlation of B/A ratios with MW for all 7433 proteins. (b) Calibration curves for all eight gels. (c) CV distribution of B/A-calculated protein MW values. (d) Error distribution of B/A-calculated protein MW values.

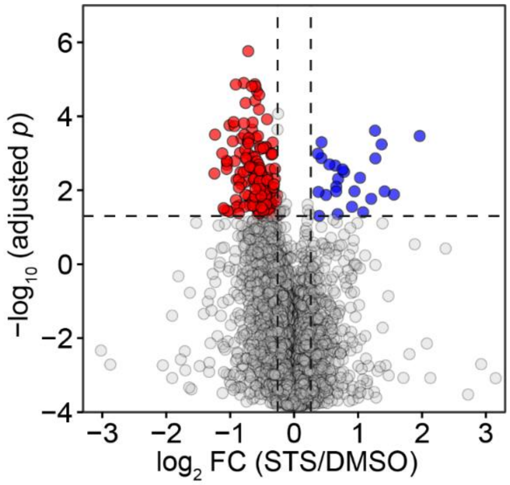

Figure S4. Volcano plot of proteins with significant MW shifts in the MS2 data set. With p values adjusted by peptides number-based multiple hypothesis correction, volcano plot shows 155 proteins significantly shift to lower MW (red) while 25 proteins significantly shift to higher MW (blue).

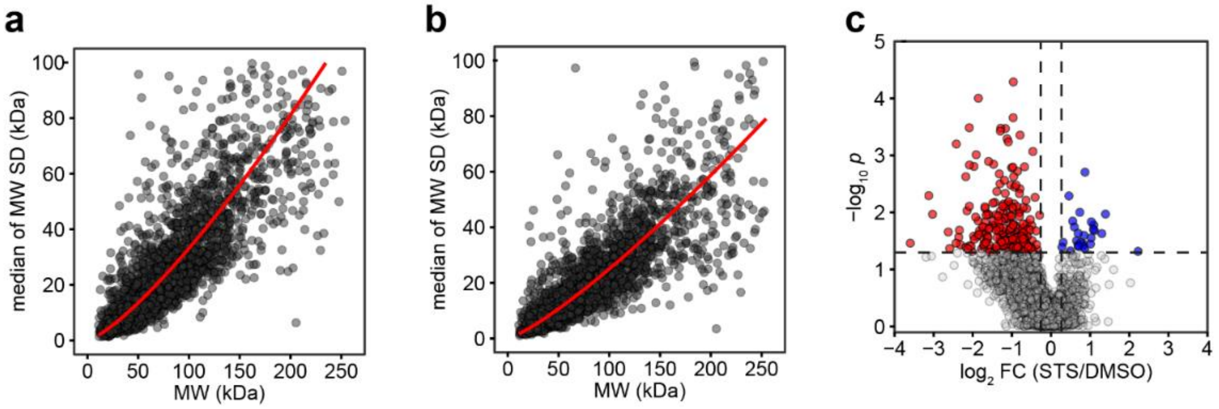

Figure S5. Standard deviation (SD) analysis for identifying caspase substrate candidates. (a) The empirical formula $SD = 0.086MW^{1.29}$ is fitted to the SD-MW plot for DMSO-treatment, with residual standard error of 13 kDa. (b) The empirical formula $SD = 0.086MW^{1.23}$ is fitted to the SD-MW plot with residual standard error 10 kDa for STS-treatment. (c) Volcano plot for 3384 proteins with ≥ 7 peptides from STS-treated HeLa cells with proteins showing significantly decreased (red) and increased (blue) SD.

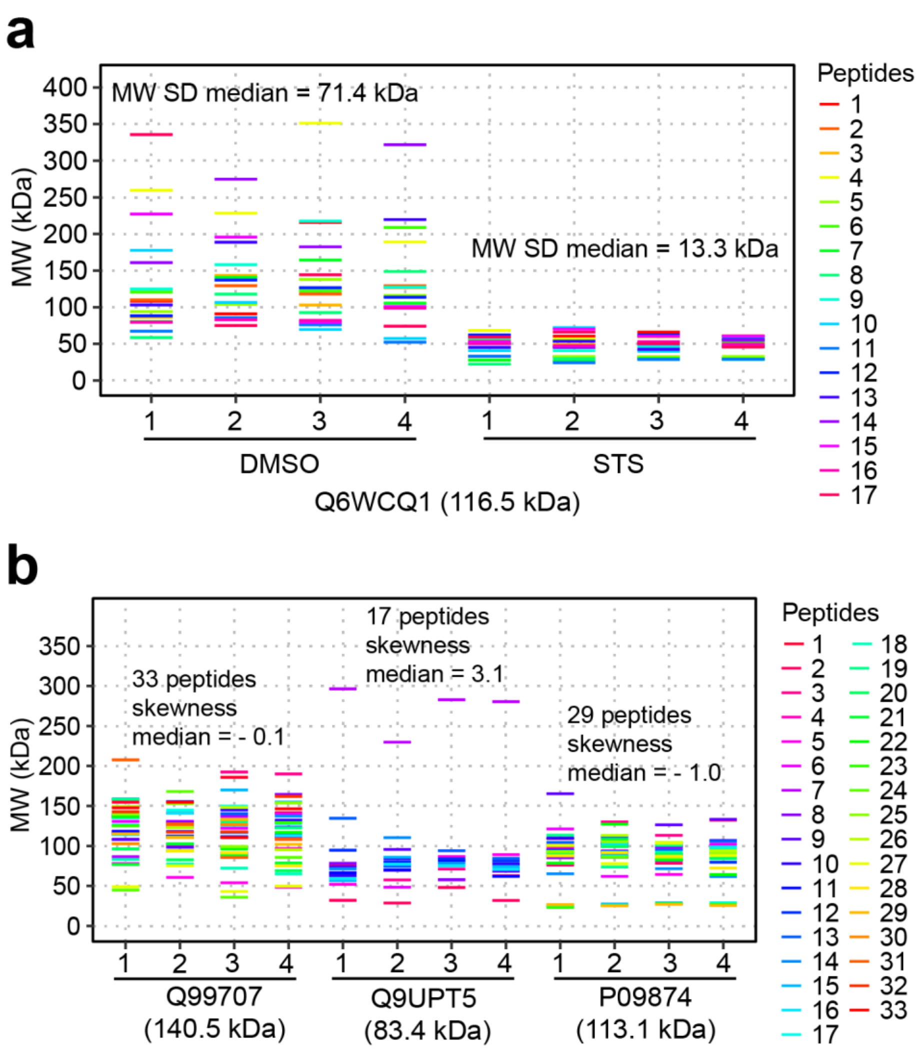

Figure S6. Protein MW estimation from peptide B/A values for SD and skewness analysis. (a) A pseudogel with one protein's MW distribution across the four replicates in DMSO- and STS-treated HeLa cells. (b) A pseudogel with peptide-derived MW estimations of three proteins in STS-treated HeLa cells.

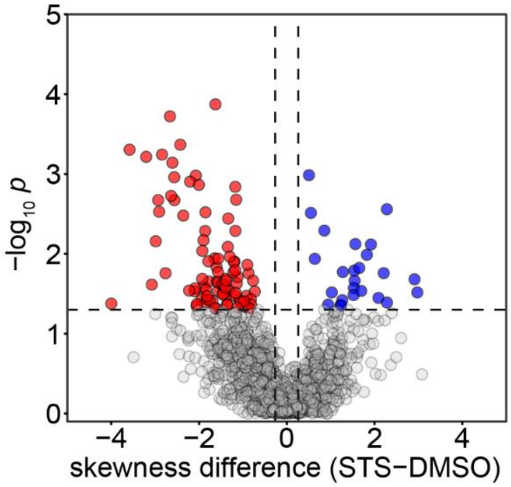

Figure S7. Skewness analysis for identifying caspase substrate candidates. Volcano plot for 1569 proteins with ≥ 13 peptides showing in STS-treated HeLa cells proteins with significantly decreased (red) and increased (blue) skewness.

Table S1. Validated caspase 3 candidate substrates. The proteins at No. 1 to 26 were found in literature. The 58 novel caspase 3 substrates were at No. 27 to 84.

No.	Protein ID	Gene names	Protein names	MW (kDa)
1	O15027	SEC16A	Protein transport protein Sec16A	251.89
2	O15042	U2SURP	U2 snRNP-associated SURP motif-containing protein	118.29
3	O43432	EIF4G3	Eukaryotic translation initiation factor 4 gamma 3	176.65
4	O60264	SMARCA5	SWI/SNF-related matrix-associated actin-dependent regulator of chromatin subfamily A member 5	121.90
5	O60271	SPAG9	C-Jun-amino-terminal kinase-interacting protein 4	146.20
6	P05783	KRT18	Keratin, type I cytoskeletal 18	48.06
7	P08670	VIM	Vimentin	53.65
8	P09874	PARP1	Poly [ADP-ribose] polymerase 1	113.08
9	P20700	LMNB1	Lamin-B1	66.41
10	P41214	EIF2D	Eukaryotic translation initiation factor 2D	64.71
11	P42166	TMPO	Lamina-associated polypeptide 2, isoform alpha;Thymopoietin;Thymopentin	75.49
12	P43243	MATR3	Matrin-3	94.62
13	P49321	NASP	Nuclear autoantigenic sperm protein	85.24
14	P51532	SMARCA4	Transcription activator BRG1	184.64
15	P78344	EIF4G2	Eukaryotic translation initiation factor 4 gamma 2	102.36
16	Q13177	PAK2	Serine/threonine-protein kinase PAK 2;PAK-2p27;PAK-2p34	58.04
17	Q14980	NUMA1	Nuclear mitotic apparatus protein 1	238.26
18	Q14C86	GAPVD1	GTPase-activating protein and VPS9 domain-containing protein 1	164.98
19	Q2NL82	TSR1	Pre-rRNA-processing protein TSR1 homolog	91.81
20	Q5SW79	CEP170	Centrosomal protein of 170 kDa	175.29
21	Q5T200	ZC3H13	Zinc finger CCCH domain-containing protein 13	196.63
22	Q6PKG0	LARP1	La-related protein 1	123.51
23	Q92538	GBF1	Golgi-specific brefeldin A-resistance guanine nucleotide exchange factor 1	206.44
24	Q9BTE3	MCMBP	Mini-chromosome maintenance complex-binding protein	72.98
25	Q9C0C9	UBE2O	E2/E3 hybrid ubiquitin-protein ligase UBE2O	141.29
26	Q9UPT8	ZC3H4	Zinc finger CCCH domain-containing protein 4	140.26
27	A1X283	SH3PXD2B	SH3 and PX domain-containing protein 2B	101.58
28	O15031	PLXNB2	Plexin-B2	205.12
29	O60256	PRPSAP2	Phosphoribosyl pyrophosphate synthase-associated protein 2	40.93
30	O60333	KIF1B	Kinesin-like protein KIF1B	204.47

No.	Protein ID	Gene names	Protein names	MW (kDa)
31	O60524	NEMF	Nuclear export mediator factor NEMF	122.95
32	O75970	MPDZ	Multiple PDZ domain protein	221.62
33	O95197	RTN3	Reticulon-3	112.61
34	P04843	RPN1	Dolichyl-diphosphooligosaccharide--protein glycosyltransferase subunit 1	68.57
35	P08237	PFKM	ATP-dependent 6-phosphofructokinase, muscle type	85.18
36	P25705	ATP5A1	ATP synthase subunit alpha, mitochondrial	59.75
37	P26358	DNMT1	DNA (cytosine-5)-methyltransferase 1	183.16
38	P27708	CAD	CAD protein;Glutamine-dependent carbamoyl-phosphate synthase;Aspartate carbamoyltransferase;Dihydroorotase	242.98
39	P30876	POLR2B	DNA-directed RNA polymerase II subunit RPB2	133.90
40	P31751	AKT2	RAC-beta serine/threonine-protein kinase	55.77
41	P46087	NOP2	Probable 28S rRNA (cytosine(4447)-C(5))-methyltransferase	89.30
42	P51114	FXR1	Fragile X mental retardation syndrome-related protein 1	69.72
43	P51858	HDGF	Hepatoma-derived growth factor	26.79
44	Q02952	AKAP12	A-kinase anchor protein 12	191.48
45	Q05209	PTPN12	Tyrosine-protein phosphatase non-receptor type 12	88.11
46	Q08AD1	CAMSAP2	Calmodulin-regulated spectrin-associated protein 2	168.09
47	Q12904	AIMP1	Aminoacyl tRNA synthase complex-interacting multifunctional protein 1;Endothelial monocyte-activating polypeptide 2	34.35
48	Q13905	RAPGEF1	Rap guanine nucleotide exchange factor 1	120.55
49	Q14126	DSG2	Desmoglein-2	122.29
50	Q14160	SCRIB	Protein scribble homolog	174.91
51	Q14669	TRIP12	E3 ubiquitin-protein ligase TRIP12	220.43
52	Q14692	BMS1	Ribosome biogenesis protein BMS1 homolog	145.81
53	Q5JSH3	WDR44	WD repeat-containing protein 44	101.37
54	Q5JSZ5	PRRC2B	Protein PRRC2B	242.96
55	Q5T5Y3	CAMSAP1	Calmodulin-regulated spectrin-associated protein 1	177.97
56	Q5TAX3	ZCCHC11	Terminal uridylyltransferase 4	185.16
57	Q5VT25	CDC42BPA	Serine/threonine-protein kinase MRCK alpha	197.30
58	Q641Q2	FAM21A	WASH complex subunit FAM21A	147.18
59	Q68E01	INTS3	Integrator complex subunit 3	118.07
60	Q6WCQ1	MPRIP	Myosin phosphatase Rho-interacting protein	116.53
61	Q6XZF7	DNMBP	Dynamin-binding protein	177.35
62	Q7RTP6	MICAL3	Protein-methionine sulfoxide oxidase MICAL3	224.29
63	Q7Z3T8	ZFYVE16	Zinc finger FYVE domain-containing protein 16	168.90

No.	Protein ID	Gene names	Protein names	MW (kDa)
64	Q7Z4S6	KIF21A	Kinesin-like protein KIF21A	187.18
65	Q8N3D4	EHBP1L1	EH domain-binding protein 1-like protein 1	161.85
66	Q8NEB9	PIK3C3	Phosphatidylinositol 3-kinase catalytic subunit type 3	101.55
67	Q8WWI1	LMO7	LIM domain only protein 7	192.69
68	Q92576	PHF3	PHD finger protein 3	229.48
69	Q96BP3	PPWD1	Peptidylprolyl isomerase domain and WD repeat-containing protein 1	73.57
70	Q96PC5	MIA2	Melanoma inhibitory activity protein 2	159.83
71	Q9BQG0	MYBBP1A	Myb-binding protein 1A	148.85
72	Q9BSJ8	ESYT1	Extended synaptotagmin-1	122.85
73	Q9C0B5	ZDHHC5	Palmitoyltransferase ZDHHC5	77.54
74	Q9H501	ESF1	ESF1 homolog	98.80
75	Q9NR30	DDX21	Nucleolar RNA helicase 2	87.34
76	Q9NS87	KIF15	Kinesin-like protein KIF15	160.16
77	Q9NSE4	IARS2	Isoleucine--tRNA ligase, mitochondrial	113.79
78	Q9NTI5	PDS5B	Sister chromatid cohesion protein PDS5 homolog B	164.67
79	Q9NUY8	TBC1D23	TBC1 domain family member 23	78.32
80	Q9UPN4	CEP131	Centrosomal protein of 131 kDa	122.15
81	Q9UPQ0	LIMCH1	LIM and calponin homology domains-containing protein 1	121.87
82	Q9UQE7	SMC3	Structural maintenance of chromosomes protein 3	141.54
83	Q9Y2D5	AKAP2	A-kinase anchor protein 2	94.66
84	Q9Y4W2	LAS1L	Ribosomal biogenesis protein LAS1L	83.06

METHODS

Cell Work and GAPPIS Sample Preparation

Cell culture. Human HeLa cells (ATCC) were grown at 37°C in 5% CO_2 using Dulbecco's Modified Eagle Medium (Lonza, USA) supplemented with 10% FBS superior (Biochrom) and 100 units/mL penicillin/streptomycin (Gibco). Low-number passages (<10) were used for the experiments.

Staurosporine Treatment and Cell Lysis. HeLa cells were cultured in 75 cm^2 flasks for 20 h, subsequently treated with 300 nM staurosporine (STS) or vehicle (DMSO) for 4 h in four biological replicates. Cells were washed twice with phosphate buffered saline (PBS) and lysed in M-PER Mammalian Protein Extraction Reagent (Thermo Fisher) supplemented with 1% protease inhibitors (Roche). The cellular lysates were centrifuged at 12,000 rpm for 10 min at 4°C and the soluble fraction was collected. The protein concentration in the lysate was measured using Pierce BCA kit (Thermo Fisher).

SDS-PAGE and Gel Excision. Cell lysate was diluted using M-PER Mammalian Protein Extraction Reagent for all samples to the same protein concentration. The electrophoresis was performed on NuPAGE 4-12% Bis-Tris Mini Protein Gel (Thermo Fisher) with two wells in MES Running Buffer under reduced conditions at 150 V for 60 min using the XCell SureLock system (Thermo Fisher). 100 µg of protein were loaded onto the gel for each sample. One STS-treated sample and one DMSO-treated sample were processed in the same tank. Novex Sharp Pre-Stained Protein Standard (Thermo Fisher) was used as a ladder. After electrophoresis the gels were washed and excised diagonally into two parts (A and B), after which each part of the gel was cut into 1 × 1 mm cubes and transferred into 5 mL LoBind tubes. The gel cubes were centrifuged, followed by sequential washes with ammonium bicarbonate/acetonitrile (1:1, v/v) and acetonitrile, each for 10 min. During the acetonitrile incubation, the gel pieces exhibited shrinkage and opacity. Subsequently, all liquid was removed.

Reduction and Alkylation. 25 mM of dithiothreitol (DTT) (Sigma-Aldrich) in 50 mM ammonium bicarbonate was added to fully immerse gel pieces. The samples were then incubated at 56 °C for 30 min. After cooling to room temperature (RT), any remaining liquid was removed. Following this, acetonitrile was added and incubated for 10 min at RT, after which all liquid was removed. Subsequently, 50 mM iodoacetamide (IAA) (Sigma-Aldrich) was

added, and the samples were incubated at RT in darkness for 1 h. Then acetonitrile was used to shrink the gel pieces for 10 min with subsequent liquid removal.

Trypsin Digestion. Each sample was rehydrated at 4 °C for 30 min by adding the same volume of 10 ng/µL trypsin in 50 mM ammonium bicarbonate containing 0.01% ProteaseMAX Surfactant (Promega). During rehydration, the absorbance of the solution was checked, and additional trypsin solution was added to ensure that gel pieces immersed completely. Subsequent incubation was performed for 2 h at 37°C with agitation at 200 rpm. Condensate from the tube walls was collected by centrifugation at 12000 × g for 10 s. The digestion solution with extracted peptides was transferred into new 2 mL tubes, and trypsin was inactivated by adding trifluoroacetic acid (TFA) (Sigma-Aldrich) to a final concentration of 0.5%.

TMT-labeling. Samples were cleaned up using Sep-Pak cartridges (Waters) and dried in a DNA 120 SpeedVac Concentrator (Thermo Fisher). The dried peptide samples were reconstituted in 50 mM EPPS buffer (pH 8.5). Acetonitrile (ACN) was added to achieve a final concentration of 30%. After cutting each gel lane into two pieces A and B, four replicates of DMSO- and STS-treated HeLa cells (eight samples) produced 16 sub-samples, one set of TMTpro 16plex was used for labeling these 16 sub-samples as shown in Figure 1g. TMTpro 16plex reagents (Thermo Fisher) were added at a 5:1 w/w ratio to each sample, followed by a 2 h-incubation at RT. The TMT labeling reaction was quenched by the addition of 0.5% hydroxylamine. All 16 TMT-labelled samples were combined, acidified with TFA, subjected to clean-up using Sep-Pak cartridges (Waters), and dried in a DNA 120 SpeedVac Concentrator (Thermo Fisher).

High-pH Fractionation. Peptide separation for deeper proteome analysis was carried out using an Ultimate 3000 HPLC system (Thermo Fisher) equipped with a Xbridge Peptide BEH C18 column (25 cm × 2.1 mm, particle size 3.5 µm, pore size 300 Å; Waters) operating at a flow rate of 200 µL/min. Fractionation was achieved through a binary solvent system comprising 20 mM NH_4OH in H_2O at pH 10 (solvent A) and 20 mM NH_4OH in acetonitrile (solvent B). The elution profile was programmed as follows (data for % of solvent B): an initial gradient from 2% to 23% over 42 min, followed by a rapid increase to 52% within 4 min, further elevation to 63% in 2 min, and a subsequent isocratic hold at 63% for 5 min. The elution process was monitored by UV absorbance at 214 nm. A total of 96 fractions, each containing a 100 µL aliquot, were collected. Fractions were subsequently combined in a sequential order (e.g. 1, 25, 49, 73), providing a total of 24 composite fractions.

LC-MS/MS Analysis

The 24 composite fractions were analyzed by LC-MS/MS using an Orbitrap Fusion Lumos mass spectrometer equipped with an EASY Spray Source and connected to an Ultimate 3000 RSLC nano UPLC system (all - Thermo Fisher). Injected sample fractions were preconcentrated and further desalted online using a PepMap C18 nano trap column (2 cm × 75 µm; particle size, 3 µm; pore size, 100 Å; Thermo Fisher) with a flow rate of 3.5 µL/min for 6 min. Peptide separation was performed using an EASY-Spray C18 reversed-phase nano LC column (Acclaim PepMap RSLC; 50 cm × 75 µm; particle size, 2 µm; pore size, 100 Å; Thermo Fisher) at 55°C and a flow rate of 300 nL/min. Peptides were separated using a binary solvent system consisting of 0.1% (v/v) formic acid (FA), 2% (v/v) acetonitrile (ACN) (solvent A) and 98% ACN (v/v), 0.1% (v/v) FA (solvent B) with a gradient of 3−28% B in 115 min, 28−40% B in 5 min, 40−95% B in 5 min. Subsequently, the analytical column was washed with 95% B for 5 min before re-equilibration with 4% B.

To identify and quantify TMT-labelled peptides, we utilized both the MS2 and SPS MS3 methods. In the MS2 approach, a full MS (MS1) spectrum was first acquired in the Orbitrap analyzer with mass-to-charge ratio (m/z) range from 375 to 1500, nominal resolution 120,000, automated gain control (AGC) target 4×10^5, and maximum injection time of 50 ms. The most abundant peptide ions were automatically selected for subsequent MS/MS (MS2) analysis with a minimum intensity threshold of 2.5×10^4 and a 30 s dynamic exclusion time. MS2 spectra were acquired in the Orbitrap analyzer with the following settings: quadrupole isolation window 1.2 Th, AGC target 1.25×10^4, maximum injection time 120 ms, fragmentation type HCD, normalized collision energy 38%, nominal resolution 50,000, fixed first m/z 110. The number of MS2 spectra acquired per each MS1 spectrum was determined by setting the maximum cycle time for MS1 and MS2 spectra to 3 s (using a top speed mode).

In the MS3 approach, we employed a synchronous precursor selection (SPS) MS3 method in a data-dependent mode. The scan sequence commenced with the acquisition of a full MS spectrum using the Orbitrap analyzer, with m/z range from 375 to 1500, nominal resolution 120,000, AGC target 4×10^5, and maximum injection time of 50 ms. The most abundant peptide ions detected in the full MS spectrum were then selected for MS2 and MS3 analysis, with maximum cycle time set to 5 s (using a top speed mode). MS2 scans were performed in the linear ion trap with quadrupole isolation window 0.7 Th, AGC target 1×10^4, maximum injection time 35 ms, fragmentation type

CID, and normalized collision energy 30%. Following the acquisition of each MS2 spectrum, synchronous precursor selection isolated up to 10 most abundant fragment ions with an isolation window 3.0 Th for subsequent MS3 analysis. These fragment ions underwent further fragmentation by HCD with normalized collision energy 55%. Finally, the MS3 spectrum was acquired in the Orbitrap analyzer with a nominal resolution 50,000, AGC target 1 × 10^5, maximum injection time of 120 ms, and m/z range 110-500.

Data processing

The raw LC-MS/MS data were analysed by MaxQuant, version 2.2.0.0. The Andromeda search engine was employed to perform MS/MS data matching against the UniProt Human proteome database (version UP000005640_9606, 20607 human sequences). Enzyme specificity was trypsin, with maximum two missed cleavages permitted. When needed, semi-tryptic option was specified for the identification of semi-tryptic peptides in the MS2 dataset. Cysteine carbamidomethylation was set as a fixed modification, while methionine oxidation, N-terminal acetylation, asparagine or glutamine deamidation were used as a variable modification. A false discovery rate (1%) was used as a filter at both protein and peptide levels. Default settings were employed for all other parameters. Peptide quantification was executed using TMTpro 16plex.

The post-MaxQuant data analysis was the following. The TMT reporter ion abundances for each sample and replicate were normalized by the sum of these abundances. Then for each peptide we calculated the abundance ratio B/A for the two parts of each diagonally-cut gel. The protein B/A values were obtained using the median of the B/A ratios of the peptides associated with a given protein. Following this, we calculated the coefficient of variation (CV) of the B/A ratios between the four replicates of DMSO treatments.

To convert the protein B/A values into MW, we established calibration curves for each gel lane using the proteins chosen as described in the main text. Utilizing the lane-specific calibration curves, we computed the protein MW values for all four replicates of the DMSO/STS-treatment and determined the distribution of the MW errors (deviations from the calibration curve), as well as its parameters, such as the centroid and full width at half maximum (FWHM).

After converting B/A values of all identified proteins to MW using calibration curves, volcano plots were generated by calculating the MW fold change (FC) between STS and DMSO treatments and conducting Student's

t-tests for the two treatment groups. The p-value adjustment was following the Benjamini and Hochberg method, employed for multiple hypothesis adjustment [1]: the peptides were ranked in descending order according to the number of peptides attributed to each protein, and p-values were subsequently adjusted by multiplying by their respective ranks. Given the follow-up validation, false discovery rate (FDR) of ≤10% was determined to be acceptable for proteins with significant MW shifts (FDR was defined as the number of proteins with significant MW increase divided by the total number of proteins with both significant MW increase and decrease).

Data availability

The data supporting the findings of this study are available in the supplementary information files. The mass spectrometry data have been deposited in the ProteomeXchange Consortium via the PRIDE partner repository (https://www.ebi.ac.uk/pride/) with the dataset identifiers PXD049007.

REFERENCES

1 Diz, A. P., Carvajal-Rodríguez, A. & Skibinski, D. O. Multiple hypothesis testing in proteomics: a strategy for experimental work. Mol. Cell. Proteomics 10, M110.004374, doi:10.1074/mcp.M110.004374 (2011).
